# Analysis of Silver Nanoparticles in Ground Beef by Single Particle Inductively Coupled Plasma Mass Spectrometry (SP-ICP-MS)

**DOI:** 10.3390/molecules28114442

**Published:** 2023-05-30

**Authors:** Alexandre Chalifoux, Madjid Hadioui, Nesrine Amiri, Kevin J. Wilkinson

**Affiliations:** Department of Chemistry, Université de Montréal, 1375 Ave. Thérèse-Lavoie-Roux, Montreal, QC H2V 0B3, Canadamadjid.hadioui@umontreal.ca (M.H.); nesrine.amiri@umontreal.ca (N.A.)

**Keywords:** silver nanoparticles, single particle ICP-MS, nanomaterials, enzymatic extraction, alkaline hydrolysis, Proteinase K, TMAH

## Abstract

The regulation and characterization of nanomaterials in foods are of great interest due to the potential risks associated with their exposure and the increasing number of applications where they are used within the food industry. One factor limiting the scientifically rigorous regulation of nanoparticles in foods is the lack of standardized procedures for the extraction of nanoparticles (NPs) from complex matrices without alteration of their physico-chemical properties. To this end, we tested and optimized two sample preparation approaches (enzymatic- and alkaline-based hydrolyses) in order to extract 40 nm of Ag NP, following their equilibration with a fatty ground beef matrix. NPs were characterized using single particle inductively coupled plasma mass spectrometry (SP-ICP-MS). Fast sample processing times (<20 min) were achieved using ultrasonication to accelerate the matrix degradation. NP losses during the sample preparation were minimized by optimizing the choice of enzymes/chemicals, the use of surfactants, and the product concentration and sonication. The alkaline approach using TMAH (tetramethylammonium hydroxide) was found to have the highest recoveries (over 90%); however, processed samples were found to be less stable than the samples processed using an enzymatic digestion based upon pork pancreatin and lipase (≈60 % recovery). Low method detection limits (MDLs) of 4.8 × 10^6^ particles g^−1^ with a size detection limit (SDL) of 10.9 nm were achieved for the enzymatic extraction whereas an MDL of 5.7 × 10^7^ particles g^−1^ and an SDL of 10.5 nm were obtained for the alkaline hydrolysis.

## 1. Introduction

There is growing interest and use of engineered nanomaterials (ENMs) over the past few decades, with applications ranging from nanoelectronics to nanotechnology enabled agriculture [1,2,3,4]. ENMs are also being used in the food sciences to answer important challenges faced by the industry [5]. For example, nano-enabled packaging is being used to extend product shelf-life and avoid spoilage, either by improving the physical properties of the packaging itself (e.g., gas-barrier properties) or by releasing active agents that slow bacterial proliferation [6,7,8]. Nonetheless, there are currently concerns with respect to the exposure of humans to nanoparticles (NPs) through their presence in food, which have led to regulations to control their use [9]. For example, there is growing public awareness of the risks associated with TiO_2_ as a food additive (E171), as reflected by the recent rulings of the European Commission [10].

From a regulatory perspective, the widespread use of ENMs comes with its own set of challenges. Their small sizes and wide range of surface properties make their detection and characterization in complex biological matrices, including foodstuffs, a non-trivial endeavor. For inorganic nanomaterials, recent advances in SP-ICP-MS have been useful to facilitate the measurement of multiple physical parameters of the NPs, including their size distribution and concentration, and the concentration of dissolved forms of the metals. Nonetheless, the non-perturbing and quantitative extraction of NPs from food is difficult, from a regulatory perspective, given that there is no widely standardized method for NP extraction and since analysis must be verified for NP stability [11]. 

A limited number of studies have developed sample preparation protocols for the analysis of NPs in biological matrices, with an emphasis on matrix degradation approaches. Indeed, in order to avoid altering the NP, most extraction procedures use ‘soft extraction’ conditions using enzymes (e.g., Proteinase K [12,13,14], Pectinase [15], Pancreatin/Lipase [16]), or alkali (usually TMAH, tetramethylammonium hydroxide [15,17,18]) to degrade the biological matrices. A gentle approach using methanol was also investigated by Laughton et al. [19]. The authors found that the use of methanol to extract Au, CuO, and ZnO NPs from leaf tissues (organic kale, lettuce, and corn) led to more reproducible results than an enzyme-based extraction. Nonetheless, variable outcomes (i.e., NP number and size recoveries, as well as reproducibility) have been reported for the extractions, depending on the nature of the treated matrix and the target nanoparticles. For example, although Vidmar et al. [20] found similar NP recoveries from placental tissue, they opted for an enzymatic approach over an alkaline treatment due to the observation of NP aggregation when using the alkaline conditions. In contrast, studies examining the extraction of NPs from fish liver [21,22] showed that the enzymatic approach had lower recoveries, likely due to the inability of the protease (Proteinase K) to properly digest fatty tissue. These diverse findings show the complexity of analyzing NPs in biological matrices, including food, which complicates any intended use of the measurements for regulatory purposes. Finally, there is no certified reference material for NPs in food with which one can build new protocols.

Therefore, the objective of this study was to investigate the parameters that influence the recovery of metallic NPs from a high fat animal matrix consisting of an in-house prepared ground beef reference material. Ultrasound-assisted extraction, using either enzymes or TMAH, was employed to gently extract the NPs from the animal tissue. Silver nanoparticles (20 and 40 nm) were chosen as the model NP due to both their widespread use as an antimicrobial agent, in addition to their sensitivity to changes during the extraction process (e.g., agglomeration, dissolution). The investigated parameters include centrifugation, sample size, and composition of the extraction solution. Emphasis was also placed on determining the analytical limitations of the SP-ICP-MS data acquisition and the medium-term stability (5 days) of a processed sample.

## 2. Results and Discussion

Representative photos of the enzymatic and alkaline (TMAH) extractions are provided in the supplementary material (Figures S1 and S2). 

### 2.1. Optimization of NP Extraction by Enzymatic Hydrolysis

In order to optimize the extraction method from the reference material, Ag NP recoveries were determined for all experimental conditions by dividing the mass of Ag NPs obtained by SP ICP-MS by the total Ag obtained following acid digestion (Table 1). An extremely poor recovery (0.9 ± 0.8%) of the 40 nm of Ag NPs was obtained with a purely mechanical degradation of the sample matrix (ultrasonication only in Milli-Q water, without enzymes). A comparatively better recovery (15 ± 7%) was obtained by a 24 h enzymatic hydrolysis (Pancreatin/Lipase) without ultrasonication. When ultrasonication was combined with enzymatic hydrolysis, the recovery increased further to 38 ± 3%. This is a good example of the synergistic effects of ultrasound-assisted enzymatic hydrolysis, which facilitated a reasonable degradation of the sample matrix in a considerably shorter time (15 min) than conventional enzymatic methods. Lyophilization was added in order to better homogenize the samples. This had no impact (38 ± 3% vs. 39 ± 5%) on the recovery of the 40 nm of Ag NPs, when equivalent amounts of meat were enzymatically degraded (i.e., once water losses associated with the freeze-drying process were taken into account). 

No Ag was detected in the meat homogenate that did not contain a Ag NP spike (total [Ag] < 0.1 ng g^−1^ after total acid digestion and quantitative ICP-MS analysis). No detectable losses of Ag were measured due to the freeze-drying and grinding of the reference material, which was spiked with 252 ± 5 ng g^−1^ of 40 nm of Ag NPs, which would correspond to approx. 1260 ng of Ag per g in dried meat. Indeed, following the loss of water during lyophilization, the mass of meat was reduced to 20.1 ± 0.1% of its original weight and a concentration of 1216 ± 88 ng g^−1^ of Ag was determined, corresponding to a recovery of 96 ± 7%.

**Figure 1:**
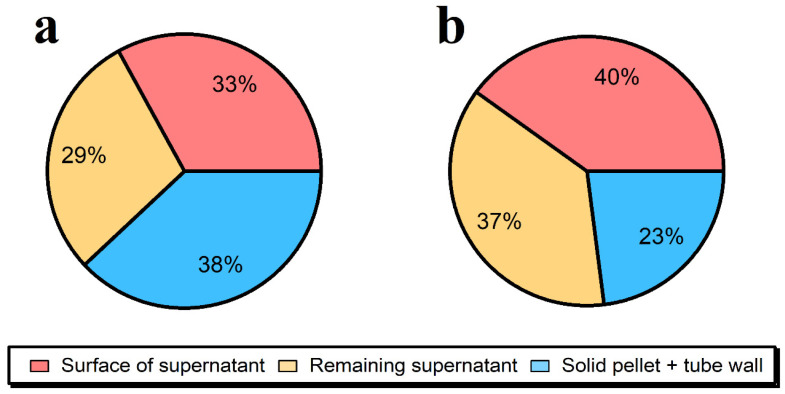
Distribution of Ag in centrifugation tubes obtained during the enzymatic extractions (1.5 mg mL^−1^ of Pancreatin + 1.5 mg mL^−1^ of Lipase, 15 min of ultrasonication) of 0.1 g of lyophilized ground beef samples after 15 min of centrifugation at 50× *g* for: (**a**) the prepared reference material at a concentration of 1216 ± 88 ng g^−1^; (**b**) a sample where the 40 nm of Ag NPs were spiked in the extraction media at a concentration of 869 ± 5 ng g^−1^, immediately before processing.

**Figure 2:**
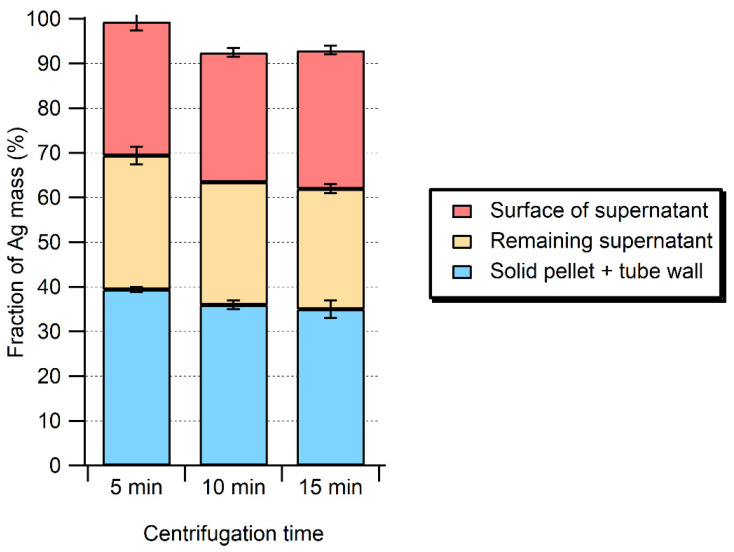
Distribution of total Ag in samples processed by enzymatic hydrolysis (Pancreatin + Lipase, 1.5 mg mL^−1^ each) as a function of centrifugation time at 50× *g*.

Mass balances were performed (Figure 1 and Figure 2) to determine where the major losses were occurring. In order to reduce the adsorptive losses as much as possible, centrifugation rather than filtration [23] was used to reduce surface interactions, while removing large agglomerates and undigested meat that could potentially obstruct the ICP-MS introduction system. The reduction in sample manipulation, such as avoiding sample transfers to ‘clean’ tubes also resulted in a much-improved extraction efficiency of 63 ± 8% Ag (from 39 ± 5%). 

A similar extraction efficiency of 62 ± 1% when using the ultrasonication-assisted Pancreatin and Lipase was used for the enzymatic hydrolysis of the ground beef that had been thoroughly equilibrated with the Ag NPs (Figure 1a). In contrast, when the 40 nm of Ag NPs were simply spiked into an extraction media containing non-spiked meat and then ultrasonicated, the extraction efficiencies were even higher (77 ± 4%), showing that part of the losses (Ag in the solid fraction that cannot be analyzed by SP-ICP-MS) occurred during the equilibration of the Ag NPs with the complex organic matrix (Figure 1b). It is hypothesized that the NPs were deeply incorporated in the meat matrix where they could not be easily analyzed. Another difficulty of the SP ICP-MS technique is that adsorptive losses of the NPs to the multiple surfaces of the ICP-MS and vessels used for sample preparation are generally inevitable given the nature of the surfaces and the fact that samples are not acidified [24]. Indeed, based upon the spike recovery experiments, adsorptive losses to tubes and ICP-MS were also substantial. Indeed, the data indicate that the observed losses (~40%) were fairly evenly split between losses to the meat and adsorptive losses to the ICP-MS and tubing.

In preliminary experiments, centrifugation times and speeds were adjusted in order to minimize the sample contact times, while increasing the separation of large particles and minimizing the losses of Ag NPs. For a centrifugation at 50× *g*, Ag was quantified in the upper supernatant, in the lower supernatant, and in the pellet for different centrifugation times (Figure 2). Most of the Ag mass was recovered in the supernatant (~60%), although substantial Ag could also be found in the meat pellet and on the inner wall of the sample tube (~40%). The centrifugation time had no impact on the distribution of Ag in the various fractions. From the insight obtained through the mass balance experiments, it became clear that adsorptive losses of the enzymatic extraction method could be reduced, but not eliminated, by reducing the sample manipulation and using polypropylene tubes [23,24]. Nonetheless, the high protein content of the enzymatic solution was likely responsible for some of the adsorptive losses. Although recoveries could be improved, in the presence of the enzyme mixtures, it was not possible to increase them above 63 ± 8% for the 40 nm of Ag NPs or 62 ± 7% for the 20 nm of Ag NPs (Table 1).

**Figure 3:**
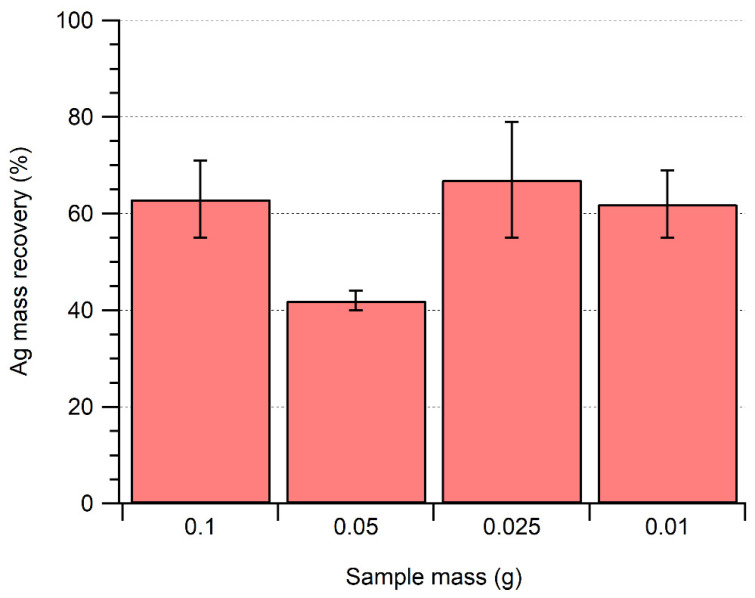
Influence of sample mass on the recovery of 40 nm of Ag NPs after enzymatic extraction of a mixture of Pancreatin (1.5 mg mL^−1^) and + Lipase (1.5 mg mL^−1^).

Further optimizations were focused on trying to improve the degradation of the meat matrix by varying the extraction volume and sample mass and examining the choice of enzymes. For an identical volume (and activity) of enzyme, the use of a smaller sample mass did not lead to a statistically significant increase in Ag NP recovery (Figure 3). Furthermore, even with smaller sample sizes, total degradation of the matrix was still not achieved, as some white-colored organic matter was visible after sample processing (Figure S1). Such a result suggests that sample degradation was limited by the nature of the sample rather than due to a mass driven limitation that would vary as a function of the mass-to-solution volume ratios. Nonetheless, the influence of the sample composition (fatty tissue content, protein composition, presence of connective tissue, etc.) on the efficiency of the extraction method has yet to be determined with certainty.

Proteinase K is a well-known enzyme, often used as the protease of choice to degrade animal tissue, often in conjunction with a surfactant such as SDS (sodium dodecyl sulfate) or Triton-X100 in the extraction medium. Indeed, data on the activity, stability, and other parameters of interest for the Proteinase K are readily available in the literature [25,26]. Nonetheless, when recoveries for the Proteinase K were compared to those obtained for porcine Pancreatin (both extractions in the presence of 1% SDS), no significant statistical difference was observed, with a recovery of ~40% in both cases. The addition of SDS to the Pancreatin extraction medium appeared to have a negative impact on the recovery of Ag NPs (39 ± 6%) when compared to the control without SDS (63 ± 3%). The surfactants are usually added in order to stabilize the NPs and prevent their agglomeration in the extraction suspensions. The decreased recovery is, thus, a counter-intuitive observation that might have resulted from an increase in the ionic strength associated with the addition of the SDS. On the other hand, homo-agglomeration of the Ag NPs was not observed with similar particle size distributions (PSDs) obtained by SP-ICP-MS in the presence and absence of SDS (mean sizes of 39.2 ± 0.2 nm vs. 39.1 ± 0.2 nm, respectively, as shown in Figure S3). Non-ionic surfactants such as Triton X100 or Tween-80 might be better suited alternatives to SDS for extractions involving low Ag NP concentrations [27].

The Ag NP recoveries presented above are lower than what is reported in the literature for the enzymatic hydrolysis of other food matrices. For example, Peters et al.’s study [28] on the Proteinase K-based extraction of Ag NPs from chicken meat is often used as a basis for method development. In that work, the recoveries of 98 ± 7% were obtained. Nonetheless, the recoveries were validated using a 60 nm Ag NP spike that was added immediately before sample processing, with a reduction in recovery (reduction of up to 40%) noted with longer equilibrium times, again demonstrating that NPs that are well-integrated into the biological matrices are harder to recover. Another approach using pork Pancreatin and Lipase for the digestion of mollusk tissues showed recoveries of about 80% for the Ag NPs [29]. In this current study, ground beef had a considerably higher fat content than the other matrices, with fatty tissues representing about half of the biological material. This possibly includes both intramuscular and intermuscular fat due to the butchering process involved in its production. Indeed, after sample processing, some white residues were observed in the slightly cloudy white suspension, a reminder that the enzymatic digestion is less efficient in degrading fats, despite the Lipase that was included in the extraction media. In addition, similar to the known adsorption of proteins and enzymes at the surface of metallic NPs, lipids can also contribute to the formation of an eco-corona [30], which could increase adsorptive losses to the container walls and increase unrecoverable NPs bound to the undigested tissues.

### 2.2. Fate of Ag NPs after the Enzymatic Extraction 

The extraction procedure that is used must limit or avoid alteration of the physical properties of the NPs during extraction and the NPs should remain stable with time so that extracted particles are the most representative as possible of the original sample. Possible changes to the size of the Ag NPs were investigated by comparing the particle size distribution (PSD) of the suspension of Ag NPs used for the preparation of the reference materials to the measured PSD obtained after sample preparation and extraction.

**Figure 4:**
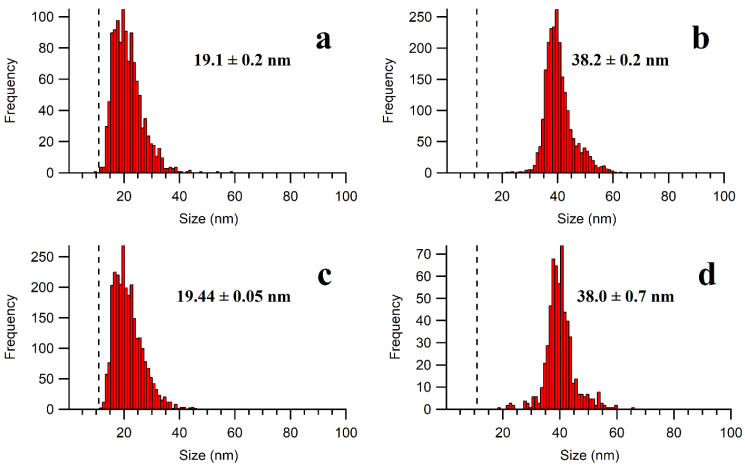
Particle size distributions of (**a**) 20 nm of Ag NPs in water; (**b**) 40 nm of Ag NPs in water; (**c**) 20 nm of Ag NPs in the enzymatic extract; and (**d**) 40 nm of Ag NPs in the enzymatic extract. A mixture of Pancreatin and Lipase (1.5 mg mL^−1^ each) was used. The calculated size limit of detection is represented by a dashed line on the PSD. The sizes correspond to the calculated mean diameters (assuming a spherical particle) of 3 samples (each measured in triplicate) with their standard deviations.

Note that in the preliminary investigations without an ice bath, the temperature of the extraction medium rose to over 70 °C following 15 min of ultrasonication, which appeared to cause a partial dissolution of the Ag NPs [31]. By immersing the extraction vessel in a water bath at room temperature, some NP dissolution was still observed with an increased detection of ionic Ag and smaller NPs, as compared to the stock suspensions. On the other hand, when an ice bath was used to limit overheating, no apparent agglomeration or dissolution was detected by the SP-ICP-MS for either the 20 nm or 40 nm of NPs extracted from the meat matrix (Figure 4). 

### 2.3. Alkaline Hydrolysis of the Meat Matrix

Given the low recoveries of the enzymatic extractions, TMAH was also tested as a means to digest the matrix, while avoiding the dissolution or agglomeration of the NPs. Indeed, the recoveries (Table 2) were significantly improved when compared to the enzymatic extraction, with a nearly complete recovery of the Ag NPs under both tested conditions. As with the enzymatic extraction, no immediate size alterations of the 40 nm NP were noted when compared to the PSD of the stock suspension (38.0 ± 0.1 nm vs. 38.2 ± 0.2 nm, Figure S4). Furthermore, upon visual observation, the obtained supernatant was clearer than the one obtained with enzymatic extraction, indicating that the alkaline extraction degraded better than the organic matrix, notably the fatty tissues, which likely explained the higher obtained recoveries. For both tested TMAH concentrations (10% and 2.5% *w*/*w* in Milli-Q water), an ionic Ag spike into the extraction media was fully recovered with no observation of Ag NP formation.

### 2.4. Stability of the Processed Samples over 4 Days

**Figure 5:**
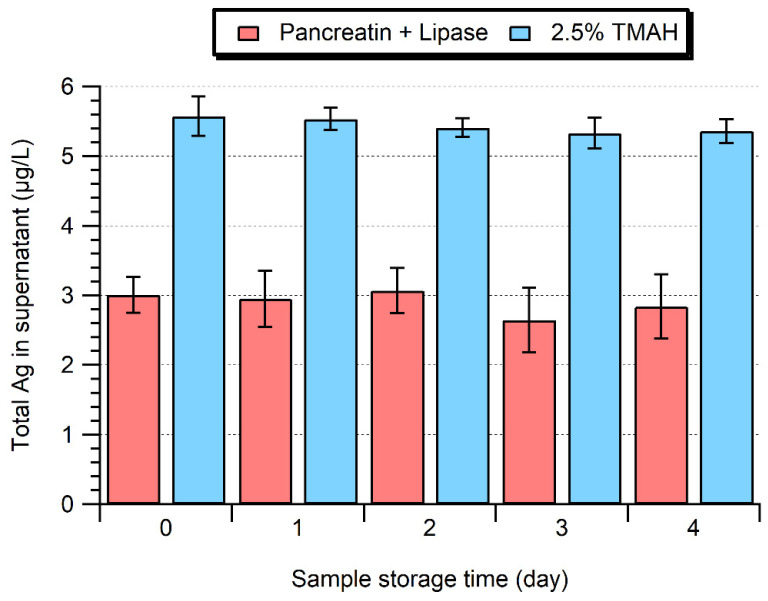
Total Ag concentration in the supernatant of extracts obtained by enzymatic (Pancreatin + Lipase) or alkaline (2.5% TMAH) extraction as a function of storage time at 4 °C.

The sample stability was examined over 4 days (Figure 5). While the cloudy white suspension obtained after enzymatic extraction eventually slowly separated into a white opaque supernatant during storage at 4 °C, it was possible to re-homogenize the sample using a vortex shaker and restore the sample to its original appearance. Ag NP sizes (Day 1: 39.1 ± 0.2 nm; Day 4: 38.8 ± 0.2 nm, Figure S5) and dissolved Ag concentrations remained stable over this time period, with no significant differences between Day 0 and Day 4 (Student’s *t*-test, *p* > 0.05). On the other hand, a partial solubilization of the Ag NPs (associated with a size decrease and increase in ionic background) was observed when storing samples at 15 °C for greater than 3 days.

Overnight settling is used in several studies as part of the sample preparation of animal tissues with TMAH [20,32,33,34]. To investigate its impact on the 10% TMAH extractions, samples were stored at room temperature in the dark for 24 h and then reanalyzed. While good recoveries (88 ± 9%) were observed for the spiked reference material, some agglomeration of the Ag NPs was observed after 24 h (Figure S6). This observation has been noted in a few other studies involving animal matrices [20,35]. Under these conditions, a loss of ionic Ag was also observed (recovery of the ionic Ag mass decreased from 89 ± 5% to 60 ± 6%), which was associated with the formation of a statistically significant (>LoQ_number_) number of small Ag NPs. The detected NPs represented a small fraction of the detected Ag (2.1 ± 0.8%), indicating adsorptive losses of ionic Ag to the inner walls of the containers or to the undigested organic matter.

**Figure 6:**
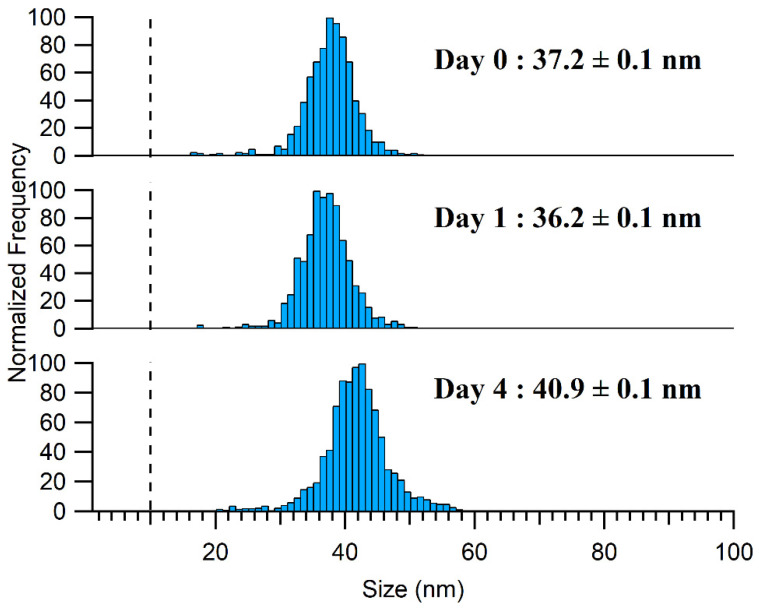
Aging of Ag NPs detected in the 2.5% TMAH extract of a processed sample stored at 4 °C with Day 0 being the day the sample was prepared. The LoD_size_ is represented by a dashed line on the PSD.

Under alkaline conditions, the sample stability was improved when a lower TMAH concentration was used in the extraction media and for samples stored at 4 °C, where they appeared to be stable for at least 24 h. Nonetheless, it should be noted that a slight increase in NP size and a slight decrease in NP concentration was observed after 4 days, indicating that agglomeration was still occurring, albeit at a reduced rate (Figure 6). For the ionic Ag spike, the samples showed good stability over 4 days following their extraction by 2.5% TMAH (Figure S7), and in contrast to the 10% TMAH extraction, the formation of Ag NPs was not observed. The lack of formation of Ag NPs might have been due to the lower ionic strength or the slightly lower pH of the medium. 

### 2.5. Evaluation of Analytical Performance

The method detection limits for the enzymatic and alkaline extractions were determined from the analysis of a processed meat sample blank (without Ag NP) prepared in the same way as the spiked reference material and analyzed ten times (Table 3, details on the calculations are included in the supplementary material). With respect to the particle size and dissolved metal concentrations, no statistical difference in the analytical performance was obtained between the two extraction media. The calculated size detection limit (LoD_size_) in the meat extracts was in the range 10 to 12 nm, which was similar to those determined in a Milli-Q water blank. Nonetheless, higher particle number detection limits (LoD_number_) were obtained following alkaline extraction. Usually, fewer than 10 NP events were detected during a 50 s acquisition time, which is on-par with the expected number of false negatives [36]. The LoD_number_ corresponded to ppt (parts per trillion) levels of particulate silver in meat. For example, under the assumption that we are detecting 20 nm or 40 nm of monodisperse Ag NPs, it is possible to calculate the particle mass LoDs of 0.21 ng g^−1^ and 1.7 ng g^−1^, respectively, for the enzymatic extraction.

## 3. Materials and Methods

### 3.1. Chemicals and Lab Equipment

Ag NP suspensions with nominal diameters of 20 nm and 40 nm (NanoXact, citrate coated) and 50 nm Au NP suspensions (Ultra-Uniform, PEG-carboxyl coated) were purchased from NanoComposix. Standard solutions of ionic Ag and Au (Inorganic Ventures) were used for calibrations, while multi-element standards (QCS-27, High Purity Standards and QCP-QCS-3, Inorganic Ventures) were used for quality control. Ultrapure grades of HNO_3_ (PlasmaPURE Plus, SCP Science, Montreal, QC, Canada), HCl (Fisher Chemicals, Hampton, NH, USA), H_2_O_2_ (30% *w*/*w*, VWR), and TMAH (25% w/w, electronic grade, Alfa Aesar #20932) were used for digestion and sample preparation. Ultrapure water (R > 18 MΩ cm; total organic carbon < 2 µg L^−1^) was generated from a Milli-Q water purification system (Millipore Sigma, St. Louis, MO, USA). Lipase from *C. rugosa* (Type VII, ≥700 U mg^−1^, lyophilized), Pancreatin from porcine pancreas (8 × USP spec., lyophilized), and SDS (sodium dodecyl sulfate, ≥99.0%) were purchased from Sigma-Aldrich, while the Proteinase K (recombinant, PCR grade, ≥600 U mL^−1^), Nylon syringe filters (25 mm disk diameter, 0.45 µm pore size), and HEPES (*N*-2-hydroxyethylpiperazine-*N*′-2-ethanesulfonate, sodium salt) were purchased from Thermo Fisher. A block digestion system (DigiPREP, SCP Science) was used for heating, when required, and ultrasonication was performed using a tip sonicator (3.2 mm tip size) on a 500 W QSonica Q500 sonicator. A Heraeus Multifuge 3S-R was used to centrifuge the samples.

### 3.2. Preparation of a Meat Reference Material

Medium ground beef (~19% *w*/*w* fat and ~19% *w*/*w* protein content, as indicated on the label) was purchased from a local grocery store. A reference material was prepared by homogenizing 50 g of fresh meat with an equal mass of Milli-Q water spiked with Ag NPs (at a concentration of ~250 ng g^−1^ with respect to the non-lyophilized meat), using a general purpose food processor. The obtained meat paste was divided into ~10 g sub-samples and kept frozen at −20 °C until use. Lyophilized meat powder was prepared by first freeze-drying the frozen meat paste for 3 days and then mixing and grinding the meat flakes into a powder using a ceramic mortar and pestle, prior to storage at −20 °C. Blank reference samples were prepared in a similar fashion without the Ag spike. The Ag content in the reference samples was monitored periodically.

### 3.3. Ultrasound-Assisted Enzymatic Hydrolysis for Ag NP Extraction

The protocol used for the extraction procedure was adapted from the work of Tabaoda-Lòpez et al. [29]. Matrix degradation and NP recovery was optimized by examining several parameters including the composition of the extraction solution (type of protease and use of surfactant), sample mass, handling of the supernatant, and centrifugation speed and time (details provided below in the Results and Discussion section).

Degradation of the fatty meat matrix was carried out by adding a precise volume (5 or 8 mL) of an extraction solution (1.5 mg mL^−1^ of protease and 1.5 mg mL^−1^ of Lipase in 5 mM HEPES buffer, pH adjusted to 7.5) to a precisely weighed aliquot of reference sample (0.01 to 0.5 g) in a 15 mL polypropylene tube followed by vortexing for 30 s. Ultrasonication was then carried out for 15 min with the sonication tip inserted ≈1.2 cm deep in a tube that was immersed in an ice bath (pulse mode: 60 s of sonication at a delivered output of 6–7 W, 10 s off).

### 3.4. Ultrasound-Assisted Alkaline Hydrolysis for Ag NP Extraction

Degradation of the meat matrix was carried out by adding 5 mL of a TMAH solution (10% or 2.5% *w*/*w* in Milli-Q water) to 0.02 g of the lyophilized reference sample, followed by 15 min of ultrasonication, using identical parameters as above. In addition to processing the reference material containing the 40 nm of Ag NPs, an aliquot of unspiked meat was processed with TMAH containing ≈10 µg L^−1^ of ionic Ag (corresponding to ≈2500 ng g^−1^ respective to the lyophilized sample mass) in order to investigate the possible formation of Ag NPs under the alkaline conditions.

### 3.5. Stability of the Processed Samples

The stability of the enzymatic or alkaline extracts were tested by analyzing the Ag content daily for 4 days after the initial sample preparation. Prior to analysis, the organic material in the extract was resuspended by vortexing the sample for 2 min followed by 3 min centrifugation at 500× *g*. A 50 µL aliquot was digested in acid (10 mL of 2% HNO_3_ for 6 h at 85 °C) and the total Ag was determined by ICP-MS. Another aliquot was sampled and diluted (1:200) for SP-ICP-MS analysis on Days 1 and 4.

### 3.6. Mass Balances of Ag NPs

The Ag NP losses during sample preparation were investigated by separating the selected enzyme processed samples into 3 fractions that were digested (Figure S8) for total Ag analysis: 4 mL of supernatant from the top of centrifuge tubes; the remaining (lower) supernatant; and Ag contained in the undigested pellet and/or adsorbed to the inner surface of the sample tubes.

### 3.7. Total Ag Determination in Samples

For solid organic samples, the total acid digestion was achieved on 0.2 g (or less) of the sample by heating samples to 85 °C for 6 h in 2 mL of Milli-Q water, 1 mL of 30% H_2_O_2_, and 1 mL of concentrated HNO_3_ in a digestion vial. The obtained solution was then diluted to 2% HNO_3_ in Milli-Q water and filtered using a 0.45 µm syringe filter prior to ICP-MS analysis. For liquid samples (from mass balance experiments), between 1 and 5 mL of the solution was digested in 2 mL of 70% HNO_3_ and 0.5 mL of 30% H_2_O_2_ for 6 h at 85 °C.

### 3.8. ICP-MS and SP-ICP-MS Sample Analysis

Unless otherwise mentioned, 3 samples were prepared for every experiment. Data acquisition was performed on a triple-quadrupole ICP-MS (NexION 5000, Perkin Elmer, Mississauga, ON, Canada). Signal intensity optimization was performed daily by adjusting the torch alignment, nebulizer gas flow, and ion optics while aspirating a multi-element ionic solution. During ICP-MS analysis, ^107^Ag isotope was monitored, while using ^115^In as an internal standard. To evaluate losses to the introduction system in the SP-ICP-MS mode, another 50 µL aliquot of every sample analyzed by SP-ICP-MS was digested for 6 h at 85 °C in 10 mL of 2% HNO_3_ before analysis for total Ag content.

For SP-ICP-MS analysis, transport efficiency was determined daily by analyzing a suspension of 50 nm of Au NPs in Milli-Q water in addition to performing an ionic calibration for Au using freshly prepared standards in 2% HCl [37]. The transport efficiencies were validated by analyzing a freshly prepared suspension of 40 nm of Ag NPs in Milli-Q water with a known NP size and concentration and an ionic Ag reference standard (QCS-27 and/or QCP-QCS-3). Triplicate SP-ICP-MS data acquisitions were performed for each sample using a 50 s scan time and 50 µs dwell time. A 5σ criteria was used to discriminate between the ionic background signal and NP signals.

For SP-ICP-MS analysis, the extract supernatants obtained after centrifugation were diluted (minimum of 1:100) in Milli-Q water. To minimize contamination and carryover between samples, the introduction system was washed with Milli-Q water for 60 s followed by 2% HNO_3_ for 90 s and Milli-Q water again for 60 s. The Syngistix Nano module (V3.2.2111.0.759, Perkin Elmer) was used to acquire and process the SP-ICP-MS data, while additional calculations of other analytical parameters that were not handled by the module (Ag NP mass concentrations and LODs) were performed manually based upon the recommendations of Laborda et al. [36]. The reported NP sizes correspond to the mean NP size determined by fitting a Gaussian distribution over a given particle size distribution (PSD).

## 4. Conclusions

Two methods were optimized and compared for the analysis of Ag NPs in medium ground beef using SP-ICP-MS. Optimization was performed using an in-house prepared reference material in order to identify the key steps that can contribute to the loss of Ag NPs. Low sample preparation times (<20 min) were achieved using ultrasonication to provide mechanical degradation of the matrix and accelerate the overall degradation of the biological tissues. The best recoveries (≈95%) and sample matrix degradation were obtained using an alkaline hydrolysis with TMAH. The enzymatic extractions were less efficient in degrading the high amount of adipose tissue in the samples, resulting in lower recoveries (≈60%). With both procedures, no immediate alteration to the NP size (aggregation, dissolution, or creation of secondary NP from dissolved Ag) was observed; however, the stability might be limited to a few days in the alkaline medium, suggesting that the samples should be analyzed as soon as possible following extraction. This study provides insights into the viable approaches to sample preparation for NP analysis involving animal matrices with relatively higher fat contents.

No difference in Ag recovery was observed when comparing two different proteases (Proteinase K and pork Pancreatin) in the enzymatic protocol. Considering the substantial difference in cost between the two enzymes, with pork Pancreatin being easily accessible in bulk quantities, it might be the enzyme of choice when involving many samples or on a limited budget.

## Figures and Tables

**Table 1:** Overview of selected extraction conditions tested for method optimization of enzymatic extraction (24 h, 37 °C). The enzymatic solution was composed of 1.5 mg mL^−1^ each of Protease (Proteinase K or Pancreatin) and Lipase. SDS = sodium dodecyl sulfate.

		Extraction			Meat Sample (Spiked with Ag NP)				Recovery of Ag Mass (%)
Extraction Medium ^a^	Sonication Time (min)	Centrifugation		Supernatant Transfer ^b^	Ag NP Size (nm)	Lyophilization	Mass (g)	Replicates	
		Speed (g)	Time (min)						
Ultrapure water only	15	50	15	Yes	40	No	0.5	3	0.9 ± 0.8
Pancreatin + Lipase	15	50	15	Yes	40	No	0.5	3	38 ± 3
	15	50	15	Yes	40	Yes	0.1	3	39 ± 5
	0 ^c^	500	3	No	40	Yes	0.1	2	15 ± 7
	15	500	3	No	40	Yes	0.1	3	63 ± 8
	15	500	3	No	40	Yes	0.025	3	67 ± 12
	15	500	3	No	40	Yes	0.01	3	62 ± 7
	15	500	2	No	40	Yes	0.02	3	63 ± 3
	15	500	3	No	40	Yes	0.05	2	42 ± 2
	15	500	3	No	20	Yes	0.02	3	62 ± 7
Pancreatin + Lipase + 1% SDS	15	500	2	No	40	Yes	0.02	3	39 ± 6
Proteinase K + Lipase + 1% SDS	15	500	2	No	40	Yes	0.02	3	43 ± 2

^a^ Extraction solution is stabilized at pH 7.5 by a 5 mM HEPES (*N*-2-hydroxyethylpiperazine-*N*′-2-ethanesulfonate) buffer in all conditions other than the ultrapure water control; ^b^ 4 mL of the upper supernatant after centrifugation was transferred to another tube; ^c^ 24 h at 37 °C without ultrasonication.

**Table 2:** Recovery of Ag from the meat reference material after alkaline extraction.

Extraction				Meat Sample		Recovery of Ag Mass (%)
Extraction Medium	Sonication Time (min)	Centrifugation		Mass (g)	Replicates	
		Speed (g)	Time (min)			
10% TMAH	15	500	2	0.02	3	92 ± 6
10% TMAH, ionic Ag spike	15	500	2	0.02	3	89 ± 5
2.5% TMAH	15	500	2	0.02	3	96.1 ± 0.3
2.5% TMAH, ionic Ag spike	15	500	2	0.02	3	114 ± 2

**Table 3:** Method limits of detection (LoD) and method limits of quantification (LoQ) determined for SP-ICP-MS analysis of Ag NPs (LoD_size_, LoQ_size_, LoD_number_, LoQ_number_) and dissolved Ag (LoD_diss_, LoQ_diss_) in two different extraction media (Proteinase K and TMAH).

		Particle Size LoD_size,_ LoQ_size_ (nm)	Particle Number Concentration LoD_number_^b^, LoQ_number_		Dissolved Metal Concentration LoD_diss,_ LoQ_diss_	
Extraction Medium ^a^			Extract/Water (×10^2^ mL^−1^)	Meat ^c^ (×10^6^ g^−1^)	Extract/Water (ng L^−1^)	Meat ^c^ (ng g^−1^)
Pancreatin + Lipase	LoD	10.9 ± 1.0	6.0 ± 2.3	4.8 ± 1.9	0.2 ± 0.2	2.0 ± 1.9
	LoQ	13.7 ± 1.2	10.9 ± 4.5	8.6 ± 3.4	0.9 ± 0.9	7.0 ± 6.8
TMAH	LoD	10.5 ± 1.7	11.5 ± 3.0	5.7 ± 1.5	0.43 ± 0.01	2.1 ± 0.1
	LoQ	13.2 ± 2.2	21.2 ± 5.4	10.6 ± 2.7	1.52 ± 0.05	7.6 ± 0.2

^a^ Uncertainties given are from 2 separate sample blanks that were prepared and analyzed 10 times each on 2 different days. ^b^ Based upon a sample blank diluted 1:200 after extraction. ^c^ Assuming a sample mass of 200 mg of lyophilized meat and a dilution factor of 1:200 prior to SP-ICP-MS analysis with an 8 mL extract volume for the enzymatic extract and 5 mL for TMAH.

## Data Availability

Raw data have been archived and are available upon request.

## Supplementary Materials

The following supporting information can be downloaded at: https://www.mdpi.com/article/10.3390/molecules28114442/s1, Figure S1: Representative images of sample preparation by the enzymatic extraction procedure; (a) before ultrasonication, (b) after ultrasonication, (c) after centrifugation, and (d) separation of colloidal lipids overtime in storage at 4 °C. Figure S2: Representative images of sample preparation by the TMAH extraction procedure; (a) untreated sample, (b) before ultrasonication, and (c) after ultrasonication and centrifugation. Figure S3: PSD of Ag NPs in enzymatic extracts (Pancreatin + Lipase) in the presence and absence of added SDS to the extraction solution. Sizes correspond to the calculated mean diameters (assuming a spherical particle) of 3 samples (each measured in triplicate) with their standard deviations. Figure S4: Particle size distributions of the 40 nm of Ag NPs in (a) water and (b) after alkaline extraction (10% TMAH) from meat matrix. The LoD_size_ is represented by a dashed line on the PSD. Figure S5: Aging of Ag NPs detected in the enzymatic extract (Pancreatin + Lipase) of a processed sample stored at 4 °C. The LoD_size_ is represented by a dashed line on the PSD. Figure S6: PSD of Ag NPs detected in samples processed with 10% TMAH and stored at room temperature for 24 h. Figure S7: Ag concentration in supernatant of samples digested in 2.5% TMAH as a function a storage time at 4 °C. Figure S8: Schematic diagram of the extraction protocol and analysis of Ag NP content in processed samples. Calculation of method limits of detection (LOD) and quantification (LOQ).
