# Supplementary material for: Analysis of Silver Nanoparticles in Ground Beef by Single Particle Inductively Coupled Plasma Mass Spectrometry (SP-ICP-MS)
Source: Molecules. 2023 May 30;28(11):4442. doi: 10.3390/molecules28114442 (PMC10254385; doi:10.3390/molecules28114442)
Supplement: Supplementary file 1 [file molecules-28-04442-s001.zip › molecules-2384285-supplementary.pdf]

molecules

MDPI

Supplementary material

Analysis of Silver Nanoparticles in Ground Beef by Single Particle Inductively Coupled Plasma Mass Spectrometry (SP-ICP-MS)

Alexandre Chalifoux[1], Madjid Hadioui[1], Nesrine Amiri[1], Kevin J. Wilkinson[1*]

[1] Department of Chemistry, Université de Montréal, 1375 Ave. Thérèse-Lavoie-Roux Montreal, QC, H2V 0B3;
* Correspondence: kj.wilkinson@umontreal.ca

Figures

Figure S1: Representative images of sample preparation by the enzymatic extraction procedure; (a) before ultrasonication, (b) after ultrasonication, (c) after centrifugation, and (d) separation of colloidal lipids overtime in storage at 4°C.

Figure S2: Representative images of sample preparation by the TMAH extraction procedure; (a) untreated sample, (b) before ultrasonication, and (c) after ultrasonication and centrifugation.

Figure S3: PSD of Ag NPs in enzymatic extracts (pancreatin + lipase) in the presence and absence of SDS added to the extraction solution. Sizes correspond to the calculated mean diameters (assuming a spherical particle) of 3 samples (each measured in triplicate) with their standard deviations.

Figure S4: Particle size distributions of the 40 nm Ag NPs in (a) water and (b) after alkaline extraction (10 % TMAH) from the meat matrix. The LoD_{size} is represented by a dashed line on the PSD.

Figure S5: Aging of Ag NPs detected in the enzymatic extract (pancreatin + lipase) of a processed sample stored at 4 °C. The LoD_{size} is represented by a dashed line on the PSD.

Figure S6: PSD of Ag NP detected in samples processed with 10 % TMAH and stored at room temperature for 24h.

Figure S7: Ag concentration in the supernatant of samples digested in 2.5 % TMAH as a function a storage time at 4 °C.

Figure S8: Schematic diagram of the extraction protocol and analysis of Ag NP content in processed samples.

Calculation of method limits of detection (LOD) and quantification (LOQ)

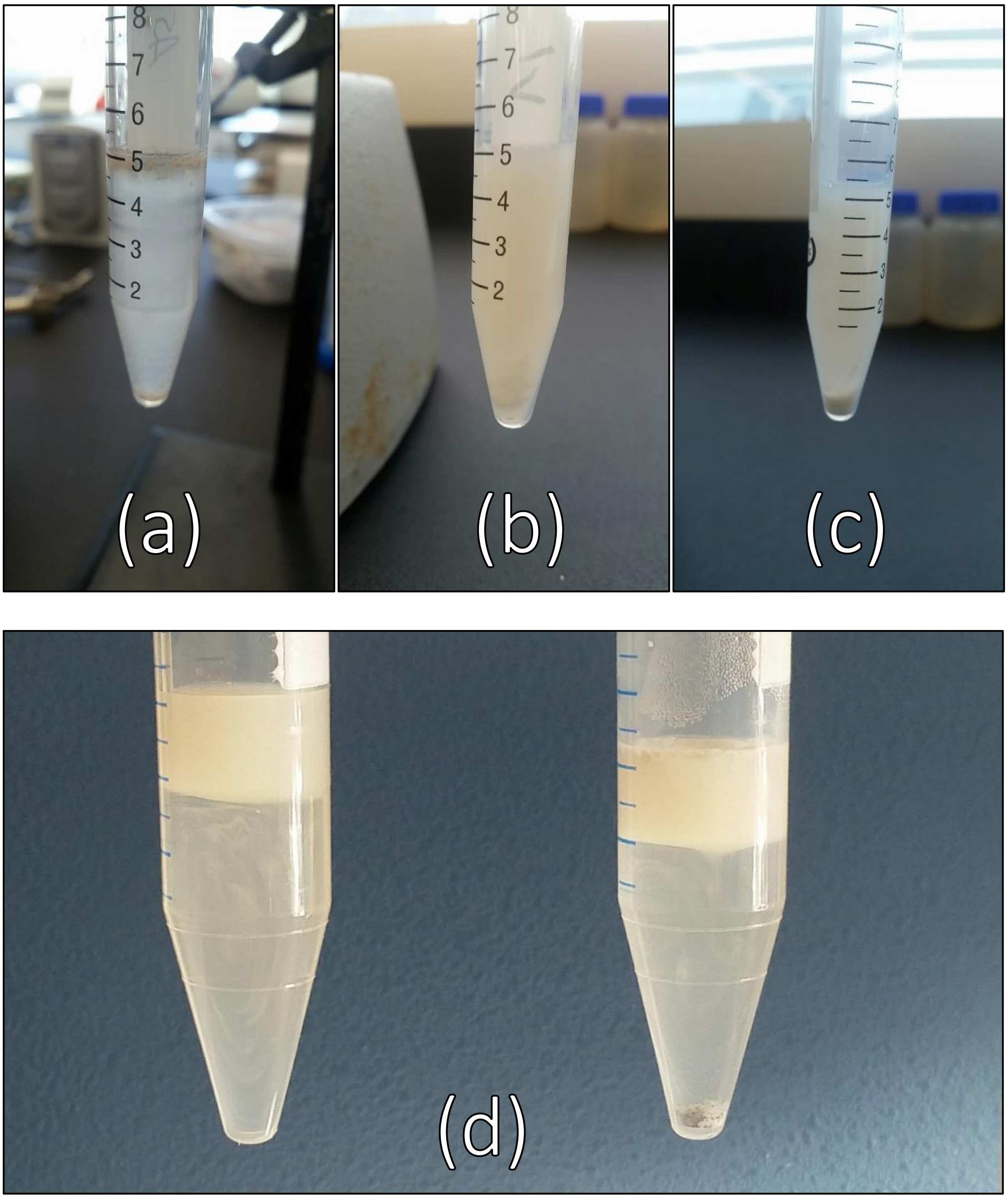

Figure S1: Representative images of sample preparation by the enzymatic extraction procedure; (a) before ultrasonication, (b) after ultrasonication, (c) after centrifugation, and (d) separation of colloidal lipids over time for storage at 4°C.

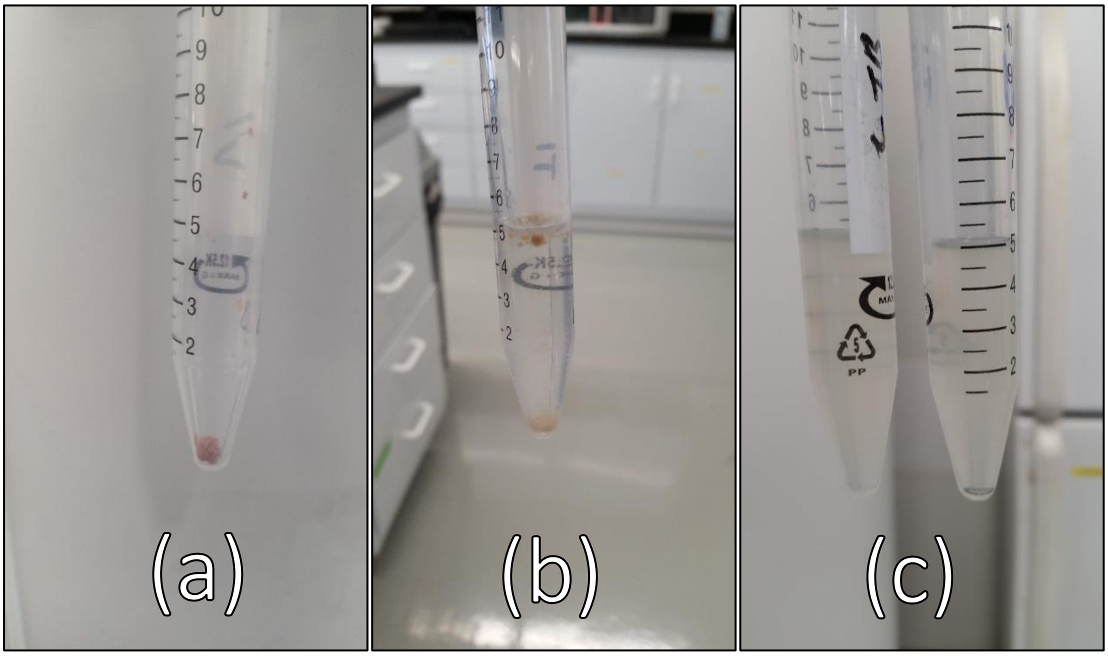

Figure S2: Representative images of sample preparation by the TMAH extraction procedure; (a) untreated sample, (b) before ultrasonication, and (c) after ultrasonication and centrifugation.

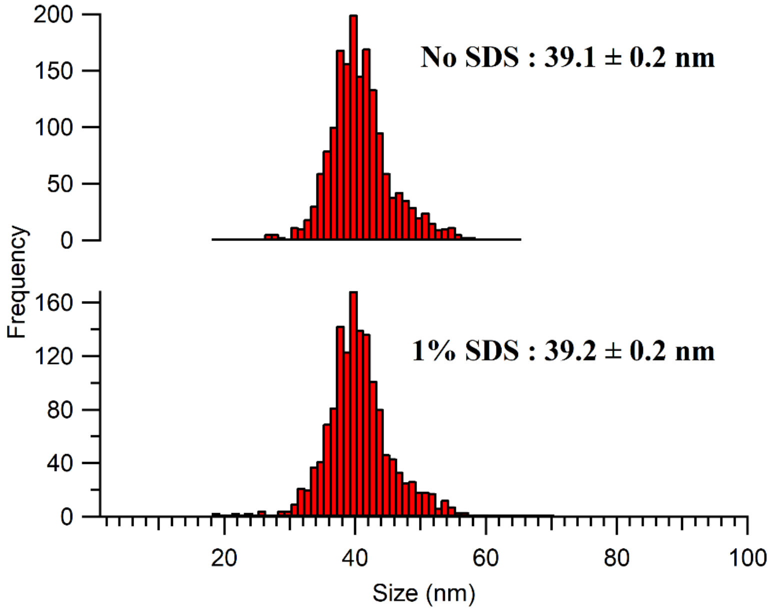

Figure S3: PSD of Ag NPs in enzymatic extracts (pancreatin + lipase) in the presence and absence of SDS added to the extraction solution. Sizes correspond to the calculated mean diameters (assuming a spherical particle) of 3 samples (each measured in triplicate) with their standard deviations.

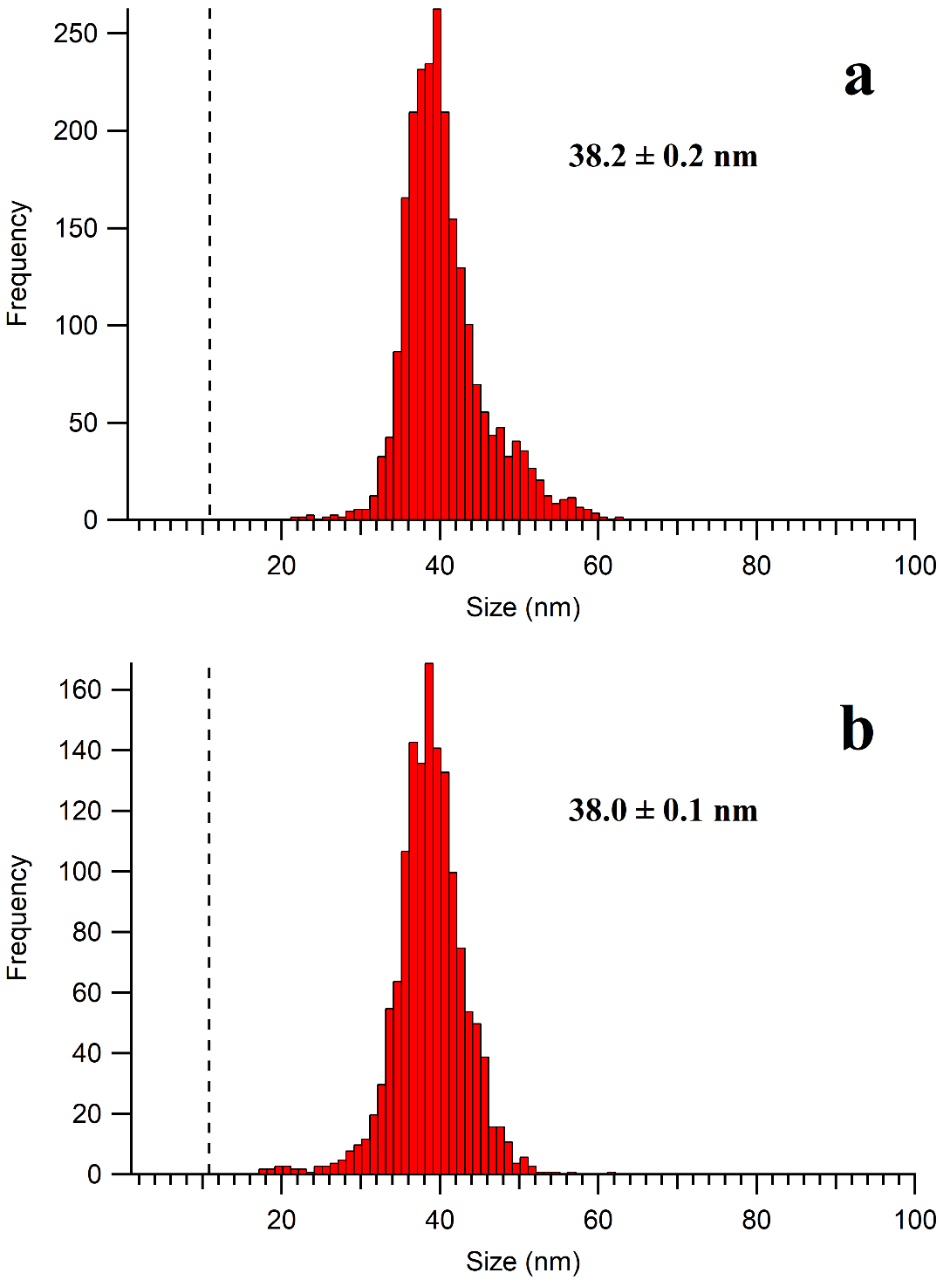

Figure S4: Particle size distributions of the 40 nm Ag NPs in (a) water; and (b) after alkaline extraction (10 % TMAH) from the meat matrix. The LoD_{size} is represented by a dashed line on the PSD.

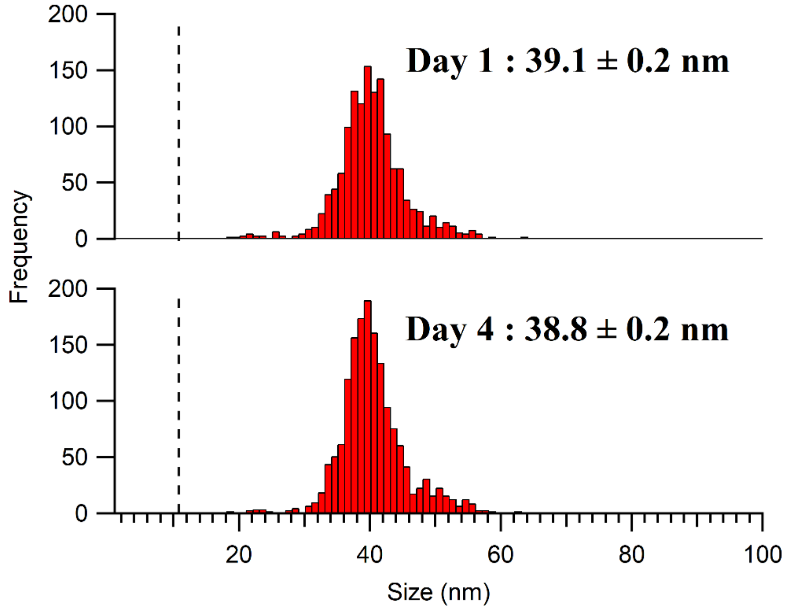

Figure S5: Aging of Ag NPs detected in the enzymatic extract (pancreatin + lipase) of a processed sample stored at 4 °C. The LoD_{size} is represented by a dashed line on the PSD.

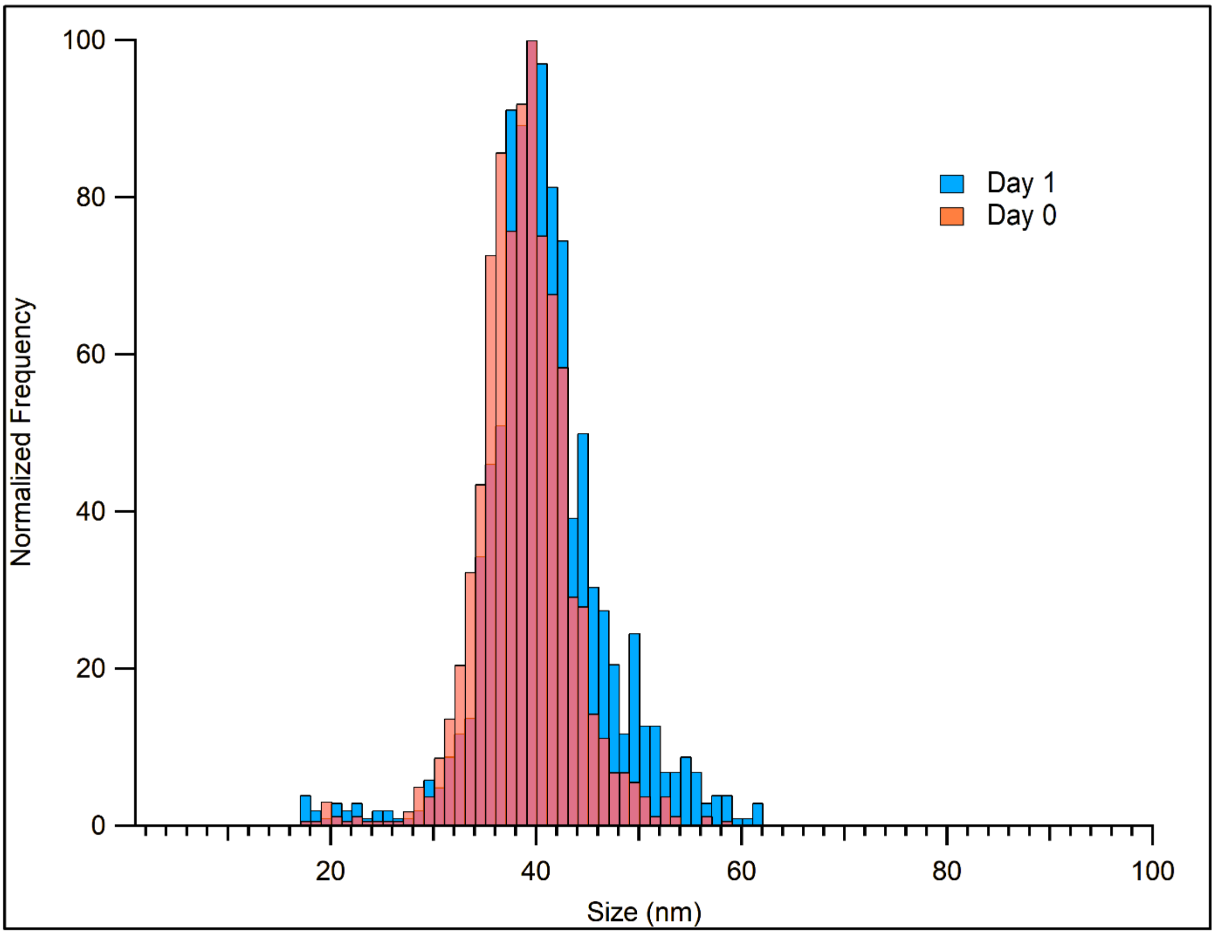

Figure S6: PSD of Ag NP detected in samples processed with 10 % TMAH and stored at room temperature for 24h.

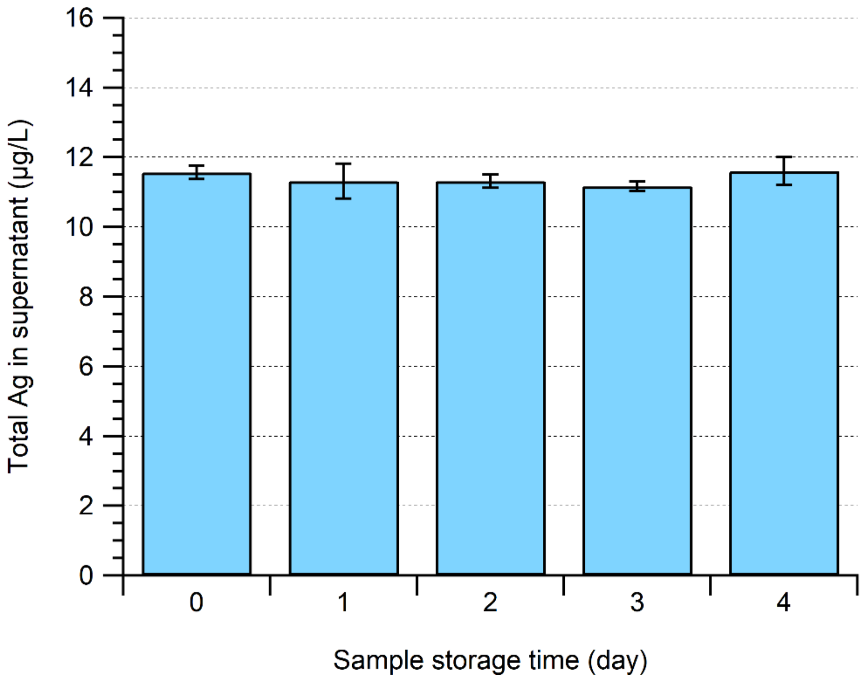

Figure S7: Ag concentration in the supernatant of samples digested in 2.5 % TMAH as a function a storage time at 4 °C.

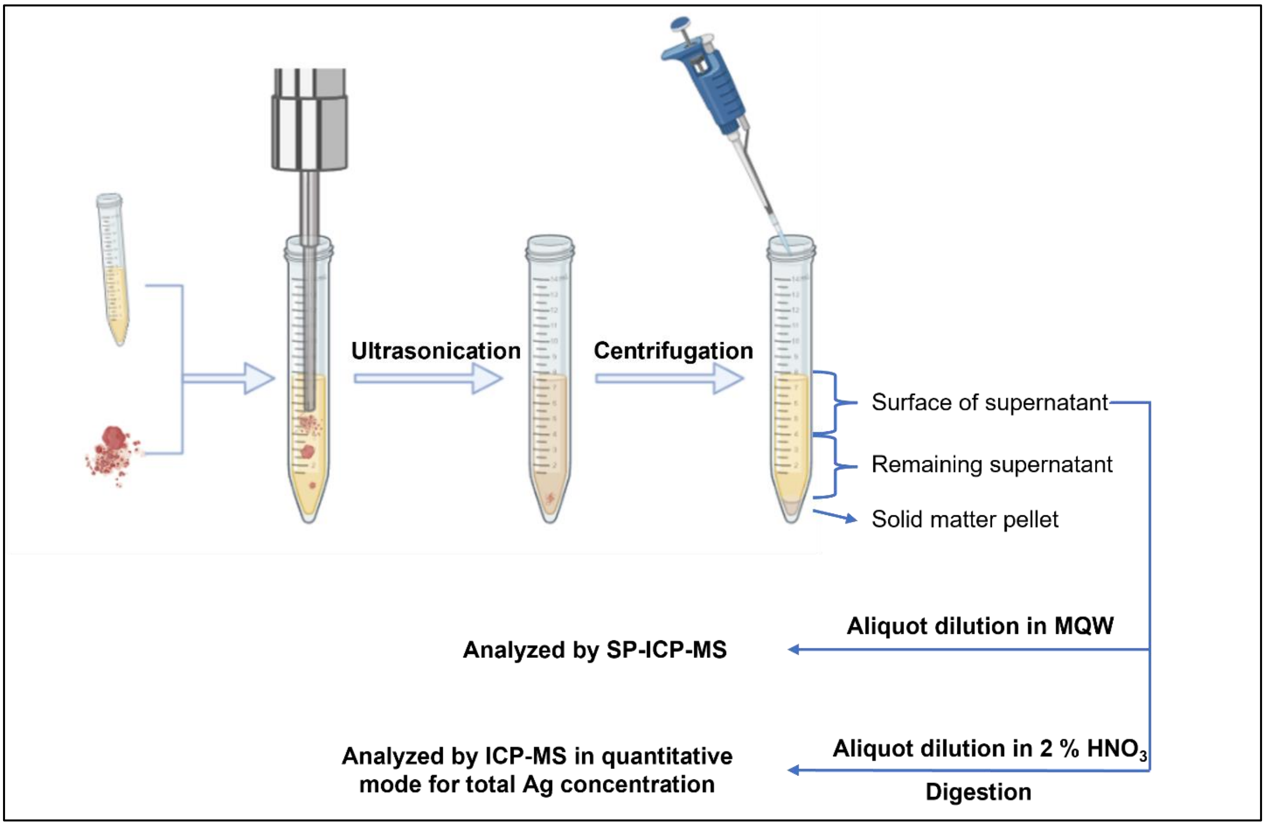

Figure S8: Schematic diagram of the extraction protocol and analysis of Ag NP content in processed samples.

Calculation of method limits of detection (LOD) and quantification (LOQ)

For further reading, equations used here are based on the works of Laborda and associates [1].

$$(1)\ LOD/LOQ_{size} = \left(\frac{6n_{\sigma}\sigma_{B}}{\frac{2}{w}\pi\rho F_{p}K_{ICPMS}K_{m}t_{dwell}}\right)^{1/3}$$

$$(2)\ LOD_{number} = \frac{5\sigma_{N,B}+3}{\eta_{neb}Q_{sam}t_{i}} \qquad (3)\ LOQ_{number} = \frac{10\sigma_{N,B}+3}{\eta_{neb}Q_{sam}t_{i}}$$

$$(4)\ LOD_{diss} = \frac{2.821\sigma_{\bar{B}}}{K_{R}t_{dwell}} \qquad (5)\ LOQ_{diss} = \frac{10\sigma_{\bar{B}}}{K_{R}t_{dwell}}$$

$$(6)\ K_{ICPMS} = \frac{K_{R}}{K_{intro}K_{m}} \qquad (7)\ K_{intro} = \eta_{neb}Q_{sam} \qquad (8)\ K_{m} = \frac{AN_{Av}}{M_{M}}$$

Where variables and constants are described as below:

n_{σ}= threshold coefficient (5 for LOD, 10 for LOQ)

σ_{B}= standard deviation of signal intensity of a blank (the average standard deviation of 10 blanks was used for calculations in this work)

w = base width of a transient particle event (400 µs used in calculations)

ρ = particle density

F_{p}= mass fraction of an element in a particle

K_{ICPMS}= detection efficiency

K_{R}= ICP-MS analytical sensitivity

K_{intro}= sample introduction factor

η_{neb}= analyte transport efficiency

Q_{sam}= sample introduction flowrate

K_{m}= element factor

A= isotopic abundance of monitored isotope

N_{Av}= Avogadro number

M_{M}= atomic mass

t_{dwell}= dwell time

$\sigma_{N,B}$= standard deviation of the number of particles events in 10 blanks

t_{i}= acquisition time

$\sigma_{\bar{B}}$= standard deviation of the mean intensity of 10 blank baselines

References:

Graphical assets from BioRender.com were used to generate part of **Figure S8**

1. Laborda, F.; Gimenez-Ingalaturre, A. C.; Bolea, E.; Castillo, J. R., About detectability and limits of detection in single particle inductively coupled plasma mass spectrometry. *Spectrochimica Acta Part B: Atomic Spectroscopy* **2020,** 169, 105883.
